# Alberta Infant Motor Scale (AIMS) Performance of Early-Term Greek Infants: The Impact of Shorter Gestation on Gross Motor Development among “Term-Born” Infants

**DOI:** 10.3390/children9020270

**Published:** 2022-02-16

**Authors:** Dimitris Syrengelas, Eirini Nikaina, Paraskevi Kleisiouni, Tania Siahanidou

**Affiliations:** 1 Department of Pediatric Physical Therapy, “Aghia Sophia” Children’s Hospital, 11527 Athens, Greece; vivikle@hotmail.com; 2 Neonatal Unit, First Department of Pediatrics, School of Medicine, National and Kapodistrian University of Athens, 11527 Athens, Greece; enikaina@gmail.com

**Keywords:** neurodevelopment, early-term, neonates, follow-up

## Abstract

Early-term birth (37^+0^ to 38^+6^ gestational weeks) may have a negative impact on infants’ neurodevelopment compared to delivery at 39 weeks or beyond. The purpose of this study was to evaluate the gross motor development of early-term infants using the Alberta Infant Motor Scale (AIMS). A total of 1087 healthy infants (559 early-term and 528 full-term infants born at 39^+0^ to 41^+6^ weeks of gestation) were studied. Mean AIMS scores were compared between the two groups at monthly intervals. The impact of gestational age on total AIMS scores was assessed by linear regression, after adjustment for chronological age, sex and SGA. Mean total AIMS scores, albeit within normal range, were significantly lower in early-term than full-term infants at the 2nd, 6th, 7th, 8th and 12th month of age; differences between groups were within three points. In multivariate regression analysis, a longer gestation by one week had a positive impact on total AIMS score during the first year of life (β = 0.90; 95% CI 0.45, 1.35). In conclusion, early-term infants exhibit worse gross motor performance during the first year of life in comparison with their full-term peers; however, the differences between the two groups are small.

## 1. Introduction

In November 2013, the American College of Obstetricians and Gynecologists and the Society for Maternal and Fetal Medicine recommended that the label “term” birth, classically defined to describe all deliveries occurring at or beyond 37^+0^ weeks of gestation, should be discouraged and replaced by the new gestational age designations; *early-term* (37^+0^ to 38^+6^ weeks of gestation), *full-term* (39^+0^ to 40^+6^ weeks of gestation), *late-term* (41^+0^ to 41^+6^ weeks of gestation) and *post-term* (42^+0^ weeks of gestation and beyond) due to lack of uniformity in neonatal outcomes [1]. Indeed, several studies have shown that early-term birth is associated with increased neonatal and infant morbidity and mortality compared with deliveries at 39 weeks of gestation or more [2,3,4]. In addition to perinatal outcomes, infants born in the early-term period are considered to be at risk for adverse long-term outcomes in several aspects, especially in neurodevelopment [3,5]. It has been reported that compared with children born full-term, early-term birth has a negative impact on cognitive [6,7] and motor development [8,9], behavior and emotional status [8,10,11], communication skills and social outcomes [8,12], school performance and academic achievements [8,13,14,15]. However, there are large variations among published studies, including methodological diversity and heterogeneity of data [16]. So far, follow-up programs for infants considered at risk for neurodevelopmental morbidity are not usually offered to the early-term infants. Given that early-term birth rates account for a significant percentage, varying from 15% to 31% among singleton live births across countries [17], further studies on outcomes of these infants are of potential clinical importance.

The Alberta Infant Motor Scale (AIMS) is a reliable, practical, observational and standardized tool for the assessment of gross motor development of infants from birth to the age of independent walking (0–19 months). Infant motor performance is assessed by AIMS in four postures: prone, supine, sitting and standing [18]. The AIMS was originally used on a Canadian population of full-term infants as a norm reference test; a re-assessment of the scale two decades later showed that the previously reported normative values remained valid [19]. Currently, the AIMS is widely used all over the world, in both clinical practice and research studies, for the assessment of motor performance in full-term infants [20]; it has also been used for the study of preterm infants, shown previously to present lower total AIMS scores up to 19 months of corrected age in comparison with infants born at term [21,22]. To the best of our knowledge, no previous studies have evaluated the gross motor development of early-term infants by AIMS.

This study aimed to assess, using the AIMS, the gross motor development of early-term infants in comparison with more mature infants born at 39 to 41^+6^ weeks of gestation.

## 2. Materials and Methods

### 2.1. Participants

Healthy Greek singleton infants, aged 0 to 19 months, who were born from 37^+0^ to 41^+6^ weeks of gestation, participated in this cross-sectional study. They were recruited from obstetric and pediatric clinics, state and private day care centers, and private pediatric offices located in different areas of the Prefecture of Attica. Demographic data based on medical birth records or maternal self-reports were recorded. Infants born preterm (<37^+0^ weeks), or with perinatal problems, neurological disorders or any acute or chronic diseases were not enrolled. The study population was categorized into two distinct groups: (a) early-term infants (37^+0^ to 38^+6^ weeks of gestation) and (b) full-term infants (39^+0^ to 41^+6^ weeks of gestation). Gestational age definition was based on medical reports of the obstetricians and neonatologists at birth. Depending on birth weight, size at birth was characterized as appropriate for gestational age (AGA) if birth weight was between the 10th and 90th percentile, or small for gestational age (SGA) if birth weight was below the 10th percentile for the corresponding gestational age and gender using the INTERGROWTH-21st growth chart [23].

In all participants, the gross motor development was evaluated once during the study period using the AIMS. Assessments were performed by the same examiner who was blinded on the infants’ gestational age at birth. The infants were assessed at their house, in obstetric and pediatric clinics or in private pediatric offices, but always with the presence of the caregiver so that the infants could feel safe and comfortable.

The purpose and design of the research was approved by the ethics committee of our institution and informed parental consent prior to enrollment was obtained.

### 2.2. Assessment Tool

The AIMS consists of fifty-eight (58) items evaluated at 4 different postures (prone: twenty-one items; supine: nine items; sitting: twelve items; and standing: sixteen items). The components assessed in each item are based on the following three elements of movement: weight bearing, posture and antigravity movements. For each item observed during the evaluation, one (1) point is credited for the infant, whereas zero (0) points are credited when the item is not observed. The sum of all items observed equals to the total raw AIMS score, which can range from zero to fifty-eight. The total raw AIMS score can be converted into a percentile rank. High percentile ranks point out maturity in gross motor development; on the contrary, infants scoring below the 5th percentile are at high risk of gross motor delay [18,19]. The AIMS has previously been standardized for use in Greek infants [20].

### 2.3. Statistical Analysis

Mean total raw AIMS score and sub-scores at prone, supine, sitting and standing posture were compared at monthly intervals between early-term and full-term infants in order to identify any differences in gross motor development between the two groups. The Shapiro–Wilk test was applied to assess normality. Comparisons were performed using the Student’s *t*-test or the Wilcoxon rank-sum test, as appropriate. The percentile rank of total AIMS score was also assessed and the number of infants exhibiting total AIMS scores below the 5th percentile was compared between the two study groups. The chi square test was used for comparison of categorical variables. Multiple regression analysis was applied to evaluate the effect of early-term birth on AIMS scores after adjusting for chronological age, sex and size at birth (SGA vs. AGA). *p*-value ≤ 0.05 was considered statistically significant. The data were analyzed using the STATA 13 and IMP SPSS 25.0 (SPSS Inc., Chicago, IL, USA) statistical packages.

## 3. Results

A total number of 1087 infants participated in this study. Of them, 559 infants were early-term and 528 infants were full-term, born at 39 weeks of gestation or beyond. Mean ± SD birth weight was 3140 ± 352 g and 3387 ± 382 g in the early-term and full-term group, respectively (*p* < 0.001). There was no difference in gender distribution between the early-term (males/females: 315/244) and full-term group (males/females: 298/230) (*p* = 0.513). In addition, the number of infants being born SGA (10 in the early-term group vs. 9 in the full-term group) did not differ significantly between the two study groups (*p* = 0.445).

Mean ± SD values of total AIMS raw scores were significantly lower in early-term than full-term infants at 2, 6, 7, 8 and 12 months of age (Table 1).

Significant differences were also observed between groups in the AIMS subscales’ raw scores (Table 2). More specifically, AIMS scores were significantly lower in early-term than full-term infants in prone posture at 6, 7 and 8 months of age; in sitting posture at 7 and 12 months of age; and in standing posture at 8 and 12 months of age (Table 2).

However, after the first year of life, no significant differences were found in total AIMS scores or sub-scores between the two study groups. Moreover, the number of infants exhibiting total AIMS scores below the 5th percentile for age did not differ significantly between the early-term and full-term group (15 and 13 infants, respectively, in the whole sample; *p* = 0.703).

Linear regression was applied following stratification by age (≤12 months vs. >12 months). After adjusting for age, gender and size at birth, early-term birth was found to be associated with a statistically significant reduction in total AIMS score but only in the group of ≤12 months of age (β = −1.56; 95% CI: −2.32, −0.79). Chronological age was an independent predictor of total AIMS score for both groups: ≤12 months (β = 4.67; 95% CI: 4.54, 4.80) and >12 months of age (β = 0.45; 95% CI: 0.30, 0.59) (Table 3). When gestational age was evaluated instead of early-term birth as an independent variable in the regression models, a longer gestation by one week was found to have a significant positive impact on total AIMS score during the first year of life (β = 0.90; 95% CI: 0.45, 1.35; *p* < 0.001).

Regression models were also run for the sub-components of AIMS at prone, supine, sitting and standing posture. After adjusting for age, gender and size at birth, early-term birth was recognized as an independent predictor for prone (β = −0.81; 95% CI: −1.18, −0.43; *p* < 0.001), sitting (β = −0.31; 95% CI: −0.54, −0.08; *p* = 0.009) and standing sub-scores (β = −0.33; 95% CI: −0.62, −0.03; *p* = 0.03) in the group of infants aged ≤12 months of age.

## 4. Discussion

Recent research has challenged whether babies born at 37^+0^ through 38^+6^ weeks of gestation share common clinical characteristics and outcomes with infants born at 39 weeks of gestation or beyond. Several studies have demonstrated a higher frequency of short and long term morbidity in early-term infants [3,4,5]. Neurodevelopmental outcomes of those infants are now being closely studied and initial reports focusing on cognition, school performance and behavioral problems demonstrate delays and deviations of the early-term infants as compared to the full-term infants [7,8,9,11,13,14,15,17]. Epilepsy and sensorineural defects have also been shown to be related to early-term deliveries [24].

However, differences in motor function between early-term and full-term infants have been less studied. It has been reported that early-term birth is an independent risk factor for cerebral palsy [24], whereas Schonhaut et al. showed that early-term infants, assessed by the Ages and Stages Questionnaire (ASQ) at 8–18 months of age, had a higher risk in developing gross motor function delays compared to their mature counterparts [25]. In another study, in infants born between 35 and 41 weeks of gestation and were assessed by ASQ during the first two years of life, a shorter gestational age was a significant predictor for fine motor skill delays [12]. Furthermore, using the Bayley scale, Rose et al. showed that for each additional week between 37 and 41 weeks of gestation, the Mental Development Index (MDI) increased by 0.8 points and the Psychomotor Development Index (PDI) increased by 1.4 points [26]. Espel at al also demonstrated that the longer the gestational length, in infants born between 37 and 41 weeks of gestation, the higher the scores on Bayley scales for both mental and motor development during the first year of life [27]. On the contrary, Wu et al. showed that early-term birth was associated with increased risk for neurodevelopment delay in the PDI, but not in the MDI domain, as measured by Bayley scales at 2 years of age [9]. To the best of our knowledge, this is the first study using the AIMS to compare the motor function between early-term and full-term infants.

In our study, significant differences in total AIMS scores between early-term and full-term infants were found practically at two time points (at 6–8 months and at 12 months of age) that normally coincide with important milestones’ achievement, namely independent sitting and standing, respectively. At 6, 7 and 8 months of age, the sub-scores for prone posture were mostly affected, also influencing the total AIMS scores. At 12 months of age, differences in sub-scores at sitting and standing posture contributed to the lower total AIMS score in early-term infants. The observed differences in motor performance between groups may be due to differences in trunk control development between early-term and full-term infants. Recent research has linked the segmental trunk control levels with gross motor performance and demonstrated that preterm infants exhibit a delay in trunk control development as compared to full-term infants [28,29]. Further studies are needed to examine whether that is also true for early-term infants.

Suboptimal gross motor performance in our early-term healthy infants, without any perinatal morbidity or complications after birth, may be explained by the interruption of the normal brain maturation process in utero. It has been shown previously that the weight of human fetal brain increases linearly with gestational age, even during the last few weeks of full-term gestation [30]. Thus, disruption of normal intrauterine brain development and exposure of the immature brain of early-term infants to a harmful environment may have adverse consequences. Interestingly, a longer gestation at birth, even within the range of 37 to 41 weeks, has been positively associated with gray matter density in brain MRI during childhood [31].

In our study, although significant differences were found in motor development between the early-term and full-term group at specific months of age, the mean values of AIMS scores at those months in early-term infants were well above the cut-off of the 5th percentile, thus within the normal range. Moreover, the number of infants exhibiting total AIMS scores below the 5th percentile for age did not differ significantly between groups. Furthermore, the observed differences in mean total AIMS scores between the two groups of infants, although significant, were rather small (within three points). As we do not have, at present, the minimal clinically significant difference of the AIMS, the clinical interpretation of the three-point difference is unknown.

The major strength of our study is the relatively large sample size that augments the power of the results. However, although more than a thousand infants were studied, the number of infants was rather small in some age categories (i.e., the age group 0–1 month). Moreover, the results of this study should be considered in light of some methodological limitations. Gestational age at birth was assessed retrospectively based on medical reports of the obstetricians and neonatologists at birth. However, it should be mentioned that obstetricians in Greece follow the guidelines issued by medical societies, thus we expect that the results are not biased. Furthermore, it was not always reported whether the delivery was spontaneous, induced or acute-prolonged. Although we only included healthy infants in the study, such information would place some of our participants at greater risk for unfavorable neurodevelopmental outcomes. Additionally, although we adjusted for infants’ gender and SGA, other potential confounders of neurodevelopment (maternal education, parity, breastfeeding, etc.) were not recorded; adjustment for those characteristics and socioeconomic circumstances known to affect neurodevelopment should be performed in future research. Finally, as this is a cross-sectional study, participants were assessed only once. However, in an attempt to more precisely evaluate the differences in motor development between groups, individual time trajectories for a long time period would possibly be more appropriate.

## 5. Conclusions

In this study, we were able to demonstrate that early-term infants exhibit worse gross motor performance during the first year of life in comparison with their full-term peers. The differences between the two groups were small, but in combination with the remarkable rate of early-term births, and the evidence for additional intellectual delays in these infants, they may bring about a significant risk for this population. Efforts should be made for the prevention of early-term deliveries, as far as possible, and for the early detection of any neurodevelopmental disorders in these infants, so that early and appropriate interventions are offered.

## Figures and Tables

**Table 1:** Total AIMS scores (mean ± SD) in early-term and full-term infants according to monthly age level.

	Early-Term (N = 559)		Full-Term (N = 528)		
Monthly Age Level	N	AIMS Score	N	AIMS Score	Score Difference (95% CI)
0 to <1	12	5.0 ± 1.2	14	5.0 ± 1.4	0 (−1.1, 1.1)
1 to <2	18	7.2 ± 1.5	16	8.9 ± 2.2	1.7 (0.3, 2.8)
2 to <3	22	9.2 ± 1.5	30	10.2 ± 2.2	0.9 (−0.1, 2.1)
3 to <4	36	11.8 ± 2.2	39	12.3 ± 2.8	0.5 (−0.8, 1.7)
4 to <5	22	17.3 ± 2.4	24	17.2 ± 2.7	−0.2 (−1.7, 1.4)
5 to <6	26	22.0 ± 3.0	31	24.6 ± 4.0	2.6 (0.7, 4.5)
6 to <7	67	26.5 ± 3.8	65	28.8 ± 5.3	2.3 (0.8, 3.9)
7 to <8	64	31.4 ± 4.7	54	34.1 ± 5.5	2.6 (0.7, 4.5)
8 to <9	27	37.6 ± 5.9	29	39.8 ± 6.6	2.2 (−1.2, 5.6)
9 to <10	44	44.3 ± 6.2	43	44.8 ± 6.8	0.5 (−2.3, 3.2)
10 to <11	33	49.0 ± 4.9	29	47.7 ± 6.2	−1.4 (−4.2, 1.5)
11 to <12	27	49.3 ± 4.8	25	52.2 ± 2.3	2.9 (0.7, 4.9)
12 to <13	20	53.9 ± 1.7	22	54.6 ± 1.8	0.6 (−0.5, 1.8)
13 to <14	26	56.0 ± 2.3	14	55.9 ± 1.6	−0.1 (−1.4, 1.4)
14 to <15	18	56.5 ± 2.0	22	57.5 ± 0.8	0.9 (−0.0, 1.9)
15 to <16	23	57.8 ± 0.3	13	57.8 ± 0.4	−0.02 (−0.3, 0.2)
16 to <17	22	57.6 ± 1.1	19	57.9 ± 0.3	0.3 (−0.2, 0.9)
17 to <18	21	57.9 ± 0.3	17	57.9 ± 0.2	0.03 (−0.1, 0.2)
18 to <19	31	56.8 ± 6.8	22	58.0 ± 0.0	1.2 (−1.7, 4.2)

CI; confidence interval.

**Table 2:** AIMS subscales scores (mean ± SD) in early-term and full-term infants according to monthly age level.

	Prone Sub-Score		Supine Sub-Score		Sitting Sub-Score		Standing Sub-Score	
Monthly Age Level	Early- Term	Full- Term	Early- Term	Full- Term	Early- Term	Full- Term	Early- Term	Full- Term
0 to <1	1.7 ± 0.6	1.7 ± 0.6	2.0 ± 0.5	1.7 ± 0.6	0.5 ± 0.5	0.8 ± 0.4	0.8 ± 0.4	0.9 ± 0.3
1 to <2	2.2 ± 0.6	2.5 ± 0.9	2.7 ± 0.5	2.6 ± 0.7	0.9 ± 0.3	1.0 ± 0.1	1.4 ± 0.5	1.7 ± 0.5
2 to <3	2.8 ± 0.7	3.4 ± 1.2	3.2 ± 0.6	3.4 ± 0.9	1.3 ± 0.6	1.4 ± 0.6	1.7 ± 0.4	1.9 ± 0.5
3 to <4	3.8 ± 1.1	4.7 ± 2.9	4.2 ± 0.8	4.3 ± 1.4	1.8 ± 0.7	2.1 ± 1.7	2.0 ± 0.4	2.2 ± 2.3
4 to <5	6.2 ± 1.3	6.0 ± 1.8	6.0 ± 1.3	6.1 ± 1.3	2.7 ± 0.6	2.8 ± 0.6	2.2 ± 0.4	2.2 ± 0.4
5 to <6	7.9 ± 1.5 *	9.9 ± 2.6	7.0 ± 1.4	7.7 ± 1.0	4.3 ± 1.4	4.5 ± 1.1	2.6 ± 0.4	2.5 ± 0.5
6 to <7	10.3 ± 2.0 ^#^	11.5 ± 2.8	8.1 ± 0.7	8.3 ± 0.7	5.4 ± 1.8 ^&^	6.2 ± 2.0	2.5 ± 0.5	2.7 ± 0.7
7 to <8	11.9 ± 2.5 ^&^	13.2 ± 3.0	8.4 ± 0.7	8.5 ± 0.7	8.0 ± 1.8	8.7 ± 1.8	3.0 ± 0.8 ^&^	3.5 ± 1.1
8 to <9	14.1 ± 3.0	15.2 ± 3.3	8.9 ± 0.2	8.9 ± 0.3	10.0 ± 1.3	10.4 ± 1.3	4.5 ± 2.2	5.3 ± 2.8
9 to <10	17.3 ± 2.9	17.7 ± 3.2	8.9 ± 0.3	9.0 ± 0.0	11.3 ± 0.8	11.2 ± 0.9	6.7 ± 2.9	6.9 ± 3.0
10 to <11	19.2 ± 2.6	18.4 ± 3.7	9.0 ± 0.0	8.9 ± 0.2	11.7 ± 0.6	11.5 ± 0.8	9.0 ± 2.2	8.9 ± 2.3
11 to <12	19.6 ± 2.7	20.7 ± 0.9	9.0 ± 0.0	9.0 ± 0.0	11.7 ± 0.6 ^&^	12.0 ± 0.0	9.0 ± 2.3 ^#^	10.4 ± 1.9
12 to <13	20.9 ± 0.3	20.9 ± 0.2	9.0 ± 0.0	9.0 ± 0.0	12.0 ± 0.0	12.0 ± 0.0	12.1 ± 1.7	12.6 ± 1.8
13 to <14	21.0 ± 0.0	21.0 ± 0.0	9.0 ± 0.0	9.0 ± 0.0	12.0 ± 0.0	12.0 ± 0.0	14.0 ± 2.3	13.9 ± 1.5
14 to <15	21.0 ± 0.0	21.0 ± 0.0	9.0 ± 0.0	9.0 ± 0.0	12.0 ± 0.0	12.0 ± 0.0	14.5 ± 2.0	15.5 ± 0.8
15 to <16	21.0 ± 0.0	21.0 ± 0.0	9.0 ± 0.0	9.0 ± 0.0	12.0 ± 0.0	12.0 ± 0.0	15.8 ± 0.3	15.8 ± 0.4
16 to <17	21.0 ± 0.0	21.0 ± 0.0	9.0 ± 0.0	9.0 ± 0.0	12.0 ± 0.0	12.0 ± 0.0	15.5 ± 1.1	15.9 ± 0.3
17 to <18	21.0 ± 0.0	21.0 ± 0.0	9.0 ± 0.0	9.0 ± 0.0	12.0 ± 0.0	12.0 ± 0.0	15.9 ± 0.3	15.9 ± 0.2
18 to <19	20.5 ± 2.5	21.0 ± 0.0	8.9 ± 0.3	9.0 ± 0.0	11.7 ± 1.4	12.0 ± 0.0	15.5 ± 2.5	16.0 ± 0.0

* *p* ≤ 0.001; ^#^ *p* ≤ 0.01; and ^&^ *p* < 0.05 in comparison with the full-term group.

**Table 3:** Multivariate regression analyses for total AIMS scores following stratification by age.

Dependent Variable: Total AIMS Score				
	≤12 Months		>12 Months	
Model	B Coefficient (95% CI)	*p*-Value	B Coefficient (95% CI)	*p*-Value
Early-term birth	−1.56 (−2.32, −0.79)	<0.001	−0.46 (−1.08, 0.15)	0.140
Age (months)	4.67 (4.54, 4.80)	<0.001	0.45 (0.30, 0.59)	<0.001
Gender (males)	−0.84 (−1.61, −0.07)	0.033	0.52 (−0.11, 1.14)	0.110
SGA	0.32 (−2.54, 3.19)	0.825	−0.33 (−2.69, 2.03)	0.784

R^2^ of models: 0.865 for ≤12 months; 0.128 for >12 months; *p* < 0.001 for both. CI; confidence interval.

## Data Availability

The data presented in this study are available on request from the corresponding author. The data are not publicly available due to privacy.
